# REDCapDM: An R package with a set of data management tools for a REDCap project

**DOI:** 10.1186/s12874-024-02178-6

**Published:** 2024-03-01

**Authors:** João Carmezim, Pau Satorra, Judith Peñafiel, Esther García-Lerma, Natàlia Pallarès, Naiara Santos, Cristian Tebé

**Affiliations:** 1 Biostatistics Support and Research Unit, Germans Trias i Pujol Research Institute and Hospital (IGTP), Carretera de Can Ruti, Camí de Les Escoles S/N, 08916 Badalona, Spain; 2 Biostatistics Unit, Institut d’Investigació Biomèdica de Bellvitge, Hospitalet de Llobregat, Spain; 3 Department of Basic Clinical Practice, School of Medicine and Health Sciences, University of Barcelona, Barcelona, Spain

**Keywords:** R, REDCap, Clinical data management, Query, Electronic data capture

## Abstract

**Background:**

Research Electronic Data CAPture (REDCap) is a web application for creating and managing online surveys and databases. Clinical data management is an essential process before performing any statistical analysis to ensure the quality and reliability of study information. Processing REDCap data in R can be complex and often benefits from automation. While there are several R packages available for specific tasks, none offer an expansive approach to data management.

**Results:**

The REDCapDM is an R package for accessing and managing REDCap data. It imports data from REDCap to R using either an API connection or the files in R format exported directly from REDCap. It has several functions for data processing and transformation, and it helps to generate and manage queries to clarify or resolve discrepancies found in the data.

**Conclusion:**

The REDCapDM package is a valuable tool for data scientists and clinical data managers who use REDCap and R. It assists in tasks such as importing, processing, and quality-checking data from their research studies.

## Background

Clinical studies require all protocol-related data to be recorded on Case Report Forms (CRFs). In the past, all CRFs were paper-based. At the end of the study, these forms were collected, boxed, and shipped to the study sponsor to be digitized using a single or double data entry process by trained personnel. In the late 1990s, the first digital versions of paper-based CRFs were developed and used. Electronic case report forms (eCRFs) quickly became popular because of their faster and more efficient data collection process and increased security. However, a lack of computers in some hospitals, difficulties in programming user-friendly interfaces, and high costs were barriers to widespread adoption. In the following decades, with the increase in digital literacy and the progressive digitization of hospitals, many of these barriers were overcome [1].

REDCap (Research Electronic Data CAPture) is a web application for creating and managing online surveys and databases [2]. It was developed in 2004 at Vanderbilt University. In short, REDCap has a set of tools that enables research teams to collect and store data. It has several key features, including an intuitive user interface, collaborative data access, user authentication and role-based security, real-time data validation, data audit capabilities, centralized data storage, and backup, data export and import functions. It was designed to handle multiple concurrent projects without requiring custom programming and is flexible enough to meet the diverse data collection needs of projects in a wide range of scientific disciplines. Although its main focus is on clinical research, REDCap can be used to collect virtually any type of data in any environment. One example would be the University of Washington (UW) Health Sciences Library (HSL) which uses REDCap for room reservations and statistics tracking [3].

Clinical data management (CDM) [4] is an important phase in clinical research that ensures the production of high quality, reliable, and statistically robust data from clinical studies. Data validation is the process of checking the accuracy and completeness of data according to the study protocol specifications. Queries identify discrepancies in the data and help to validate the data. Managing queries involves reviewing these discrepancies, investigating their causes, and resolving them or declaring them unresolvable [5].

R [6] is an open-source program primarily used for statistical analysis and is widely used in clinical research. R has a large community of users who develop packages for specific topics. There are a limited number of packages available to interact with REDCap data and, as far as we know, none of them offers a complete view of CDM, with functions for importing and processing data from REDCap as well as for data validation, checking programs, and discrepancy management. Therefore, we have developed the REDCapDM [7] package to facilitate clinical data management in studies that use REDCap as the electronic data capture interface and R as the data management tool.

## Implementation

The REDCapDM package has been developed in R, version 4.3.0. The version 0.9.5 of the REDCapDM package is freely available via the Comprehensive R Archive Network (CRAN) at https://cran.r-project.org/package=REDCapDM and the source code is freely available online through GitHub at https://github.com/bruigtp/REDCapDM. The REDCapDM package dependencies are dplyr [8], REDCapR [9], janitor [10], stringr [11], stringi [12], magrittr [13], tidyr [14], purrr [15], tidyselect [16], tibble [17], labelled [18], and rlang [19].

This package allows users to use REDCap data in R, either via the files in R format exported from REDCap or via an API connection. Once the data is imported to R, it can perform data transformation and data organization. It can also identify data discrepancies as missing or extreme values and facilitate the query management process. The main functions of the package are described below, and a practical example is provided in the results section.

### redcap_data

This function reads data from a REDCap project. Users can use an Application Programming Interface (API) connection without going through the interactive REDCap interface, or use exported data from REDCap in R format. In the first case, this function requires the uniform resource identifier (URI) of the REDCap project and a character vector with the token code. As an API token provides a 'back door' access to your data, this is a method that should be used with caution. In the second case, if an API connection is not used, this function requires the path where the exported data is located. In this case, the data and the dictionary must be specified via the ‘data_path’ and the ‘dic_path’, respectively. Also, if the REDCap project is longitudinal (more than one event), it’s worth filling in the 'event_path' argument in order to read the mapping of the events and the forms of the REDCap project. Other functions in the package will need the information of the event-form mapping to work properly.

### rd_transform

This function performs several data transformations. First, it recalculates all auto-calculated fields to identify any discrepancies between the recalculated values and the original ones. Then, it changes the names of the checkbox variables to the names of their options, and the name of their labels to 'No/Yes'. If the checkbox depends on a branching logic, it also handles the missing values. Additionally, it replaces the original variable names with their factor format versions, except for 'redcap_event_name' and 'redcap_data_access_group', which retain both versions, and for the checkboxes which have already been transformed. Then the branching logic expressions in the dictionary are converted from REDCap logic to R logic and the variables that contain a defined pattern (by default, the pattern is '_complete' and ‘_timestamp’) are eliminated. It also allows to change the final structure of the transformed dataset and to split it by events or by forms.

### rd_query

This function generates queries using expressions to identify discrepancies in the data. It can be used to identify missing values, values that fall outside the lower and upper limit of a variable and other types of inconsistency.

### rd_event

This function identifies missing events in the data. Data from a longitudinal REDCap project has one row per record and event, however, by default REDCap does not export the corresponding rows of events with no data collected.

### check_queries

This function compares a previous set of queries with a new one and allows the user to check which queries are new, which are pending, which are miscorrected or, conversely, which have been resolved.

## Results

We would like to illustrate the main functionalities of the REDCapDM package with an example using the built-in dataset. COVICAN is a dataset included in the package that corresponds to a partially transformed random sample of the COVICAN study [20]. COVICAN was an international, multicenter cohort study of cancer patients with COVID-19. The aim of the study was to describe the epidemiology, risk factors, and clinical outcomes of coinfections and superinfections in onco-hematological subjects hospitalized with COVID-19. The full code and further results of the following example can be found in the package vignette (https://bruigtp.github.io/REDCapDM/articles/REDCapDM.html).

The built-in COVICAN dataset was created using the *redcap_data()* function. This function allows users to import data from a REDCap project into R using the two REDCap exported files (*.R* and*.csv* file) as well as the project’s dictionary. All three files must be located in the same directory, but in order to use this function, the user only needs to specify the path to two of them, as shown in the following example:


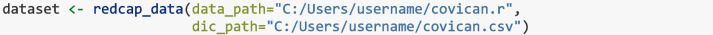


If the REDCap project is longitudinal (contains more than one event), a third element should be specified with the correspondence of each event with each form of the project. This csv file can be downloaded in ‘Designate Instruments for My Events’ within the ‘Project Setup’ section of REDCap and should be specified with the argument *event_path*.


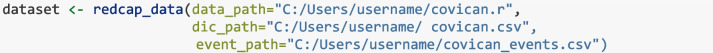


The output of the function is a list containing the data, the dictionary and the event-form correspondence (if specified) of the REDCap project read into R, which is used in the primary functions of the package. It will therefore have the same structure as the COVICAN dataset, which is described below:


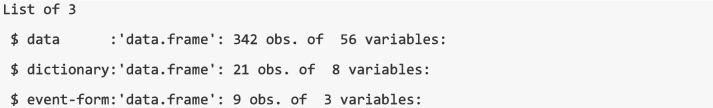


The rd_transform() function can be used to pre-process the data exported directly from REDCap and read by the *redcap_data()* function. The necessary elements that must be provided are the dataset to be transformed, the dictionary and, if the project is longitudinal, we must provide the event-form to take full advantage of the function. These elements can be specified either individually using the function arguments, or in a list with the same structure as the output of the redcap_data() function:


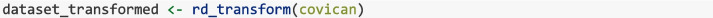


The output of this function is a list with the transformed dataset, the dictionary, and the following output of the results of each transformation step:


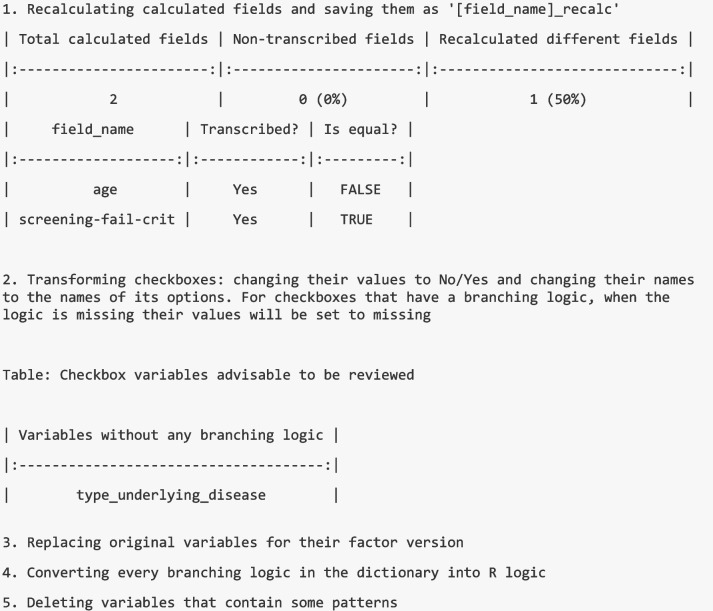


We can see that by default the basic transformation consists of five steps. The first one is to recalculate all the possible calculated fields to see if there are any discrepancies with those automatically calculated by REDCap. The calculated fields that contain certain smart variables that the function cannot translate are reported in the result trace. Looking at the trace of the results, we can see that both calculated fields could be transferred and that the recalculated values of one of the two fields (age) do not match the values of the original calculated field. The second step is to transform the checkboxes. By default, it changes the names of the checkboxes to the name of the corresponding option and the name of their labels to ‘No/Yes’. If the checkbox has a branching logic, it assigns a missing value when the result of the branching logic is also missing as these checkbox variables, by default, do not have missing values. Checkbox variables without a branching logic are reported in the result trace and it is suggested to check them to confirm if they should have a missing value. The third step is to replace the original variables with their factor version. The fourth step is to convert the branching logic expressions specified in the dictionary from REDCap logic to R logic and, finally, the fifth step is to delete, by default, the variables containing the pattern “_complete” and the pattern “_timestamp”.

Using the function’s arguments, we can modify some of these default basic steps or perform further transformations. For example, if we want the transformed dataset to be separated by event, we can specify this with the *final_format* argument:


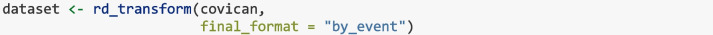


The transformed dataset in the output of the function will now be a nested data frame containing as many data frames as there are events in the REDCap project. The name of the events and the variables found in them are specified in the *events* and *vars* columns:


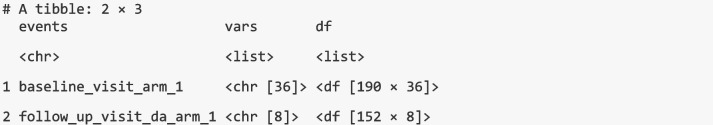


For the following functions we can use either the raw data read by the *redcap_data()* function or the pre-processed data with the *rd_transform()* function. For this example, we will use the list in which our transformed data, the associated dictionary and the event-form mapping have been stored: *dataset_transformed*.

We can use the *rd_query()* function to identify either missing values, extreme values or other types of inconsistencies. For example, if we wanted to identify missing values for variables such as chronic pulmonary disease or age, the following R code could be used:


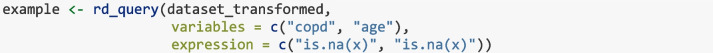


We have to specify the variables and the expression to be applied to them. The argument can either be used to specify an expression for each of the corresponding variables, or to apply the same expression to all of the variables. There is no need to specify the events where the variables are collected as the function will retrieve them automatically from the event-form mapping element.

The output of this function is a list with two elements: a data frame containing the information needed to identify each query in the REDCap project (Table 1); and a summary of the number of queries per variable and query type (Table 2).

**Table 1:** First three rows of the queries report

Identifier	DAG	Event	Instrument	Field	Description	Query	Code
100–58	Hospital 11	Baseline visit	Comorbidities	copd	Chronic obstructive pulmonary disease	The value is NA and it should not be missing	100–58-1
102–113	Hospital 24	Baseline visit	Demographics	age	Age	The value is NA and it should not be missing	102–113-1
105–11	Hospital 5	Baseline visit	Comorbidities	copd	Chronic obstructive pulmonary disease	The value is NA and it should not be missing	105–11-1

**Table 2:** Summary of queries

Variables	Description	Event	Query	Total
copd	Chronic obstructive pulmonary disease	Baseline visit	The value should not be missing	6
age	Age	Baseline visit	The value should not be missing	5

In Table 1 we can see that one of the missing values in the variable *copd* corresponds to a patient with the identification number “100–58” belonging to “Hospital 11”. It also shows that this missing value is in the instrument “Comorbidities*”* of the event “Baseline visit*”*. According to Table 2, 6 queries were identified in the *copd* variable and 5 queries in the variable *age*.

REDCapDM also allows the user to identify missing events in a dataset using the *rd_event()* function. This function was developed to overcome a limitation of REDCap, which only exports the rows corresponding to events that contain data in at least one of the collected variables. Empty events, such as a missing follow-up visits due to a patient's death, are not exported by REDCap. To identify these missing events, the following R code could be used:


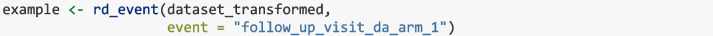


The output is identical to the previous function. It consists of a data frame that allows us to identify each missing event and a list with a summary of the total number of missing events.

This example illustrates the process of dealing with missing values in variables and missing events in our dataset. Once all the queries have been identified, the normal process would be to update the dataset in REDCap to resolve them. We could then repeat the import and query identification process using the new dataset and generate a new report. To compare this new report with the first one we had, we can use the *check_queries()* function:


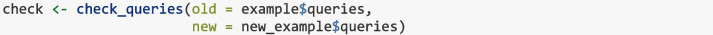


This function also returns a list with two items. The first item is a merged dataset of the two query reports with a new column indicating which of these queries are new, which are resolved, which are pending resolution and which have been miscorrected (Table 3). The second element of the list is a summary of the total number of queries grouped by the categories mentioned above.

**Table 3:** Follow-up of the identified queries

Identifier	DAG	Event	Instrument	Field	Description	Query	Code	Modification
100–58	Hospital 11	Baseline visit	Comorbidities	copd	Chronic obstructive pulmonary disease	The value is *NA* and it should not be missing	100–58-1	Pending
100–79	Hospital 11	Baseline visit	Comorbidities	copd	Chronic obstructive pulmonary disease	The value is *NA* and it should not be missing	100–79-1	New

## Discussion

A free and open-source software solution called REDCapDM has been developed to manage data from REDCap using R. This R package provides functionality for reproducible workflows that transform raw data into analysable data, with a focus on clinical research. However, REDCapDM can be used in any research study using REDCap data that requires data validation.

Nowadays, many clinical research studies collect data using electronic data capture systems, and REDCap is a popular tool for this purpose. While there are other commercial solutions available, REDCap is free to REDCap Consortium Partners and provides a secure web application with authentication and data logging. Research groups have complete autonomy and control over their projects.

REDCapDM supports clinical data management with a set of functions specifically designed for REDCap data processing. While there are other R packages available on the CRAN repository for REDCap, they are limited in number, and each has a very specific focus. To access data stored in REDCap using the Application Programming Interface (API), you can use REDCapR [9], rccola [21] and redcapAPI [22]. To create R data packages from REDCap projects, you can use REDCapExporter [23]. To convert REDCap data into tidy data frames and process them, you can use REDCapTidieR [24] and tidyREDCap [25]. To our knowledge, REDCapDM [7] is the first package developed for clinical data management in a wider scope including specific functions for importing, validating, and auditing data from REDCap.

As limitations, we should acknowledge that this package does not consider all the possible types of structures that a REDCap project may have, as it has been adapted to and tested on a limited number of different structures. We should also point out that the data pre-processing may not be able to handle complex REDCap logic involving some specific smart variables as the translation into the R language is difficult. If this problem is encountered in the branching logic of complex variables or in the calculation of calculated fields, warnings will be issued.

We plan to make some improvements to the query identification and tracking process to minimize errors and cover a wider range of possible structures. We would also like to extend the scope of the data pre-processing to cover up specific transformations of the data that may be required in some specific scenarios. As a long-term plan, we would like to complement this package with the development of a shiny application to facilitate the use of the package and make it as user friendly as possible.

## Conclusions

The REDCapDM is an R package that provides a set of useful functions for importing, organizing, and quality checking data from REDCap. It is designed to facilitate the study data management workflow, particularly in clinical research, and to help to ensure high quality and reliable data that is ready for analysis. This package fills a gap in the available tools for managing REDCap data and is a valuable resource for researchers.

## Availability and requirements

Project name: REDCapDM.

Project home page: https://cran.r-project.org/web/packages/REDCapDM/

Operating system: Platform independent.

Programming language: R

License: published under the GNU General Public License Version 2.

Any restrictions to use by non-academics: Commercial organizations are welcome to contact the author prior to use.

## Data Availability

The datasets generated and/or analyzed during the current study are available in the REDCapDM GitHub repository, https://github.com/bruigtp/REDCapDM/tree/main/data.

## Abbreviations

API: Application Programming Interface
CDM: Clinical data management
CRF: Case report form
eCRF: Electronic case report form
GLP: General Public License
GNU: GNU's Not Unix
REDCap: Research Electronic Data CAPture
